# Recurrent bullous erythema multiforme due to oral contraceptive therapy

**DOI:** 10.1097/JW9.0000000000000142

**Published:** 2024-04-02

**Authors:** Afsoon Ghafari-Saravi, Teri M. Greiling

**Affiliations:** a Department of Dermatology, Oregon Health & Science University, Portland, Oregon

**Keywords:** autoimmune progesterone dermatitis, bullous, drug-induced, erythema multiforme, oral contraceptives, progesterone

**What is known about this subject in regard to women and their families?**

- Recurrent erythema multiforme (EM) is a hypersensitivity-related skin disorder that typically manifests as distinct target-like lesions, with or without central vesicles or erosions, involving mucocutaneous sites.
- The exact etiology of EM is not well understood, but it is often triggered by infections such as herpes simplex virus, other infectious agents, or medication reactions.
- To date, there has been limited research associating bullous EM and oral contraceptive pills (OCPs).

**What is new from this article as messages for women and their families?**

- There are several reports of EM in women driven by endogenous progesterone, known as progesterone hypersensitivity or autoimmune progesterone dermatitis, which is thought to be related to cyclical rises in endogenous progesterone, often appearing several days before menses in a fertile woman with a previous history of exogenous progesterone intake and sometimes in association with pregnancy.
- In contrast, our patient had cyclical flares of bullous EM while taking exogenous desogestrel found in her OCP.
- Our case is the first reported incidence, to our knowledge, of recurrent bullous EM in association with OCPs with complete resolution upon discontinuation of OCP.

## Dear Editor,

Recurrent erythema multiforme (EM) is a hypersensitivity-related skin disorder that typically manifests as distinct target-like lesions, with or without central vesicles or erosions, involving mucocutaneous sites.^1,2^ The exact etiology of EM is not well understood, but it is often triggered by infections such as herpes simplex virus (HSV), other infectious agents, or medications.^3^ To date, there has been limited research associating bullous EM and oral contraceptive pills (OCPs).

We present a patient on a combined OCP with recurrent bullous EM, refractory to antiviral and immunosuppressive therapies, who achieved sustained remission after discontinuation of her OCP.

**Fig. 1.**
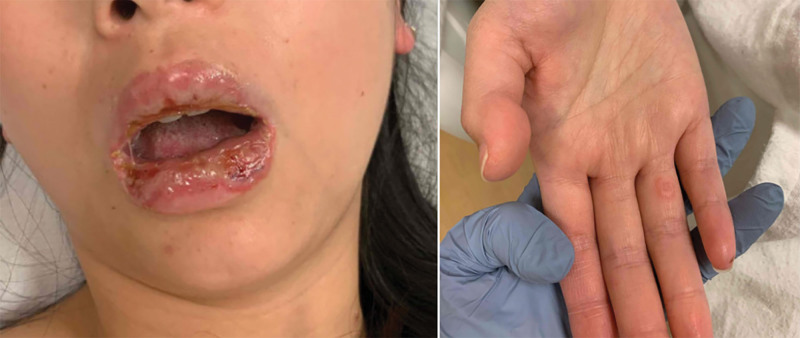
Mucosal erosions and targetoid papules at initial admission.

A 22-year-old woman without significant past medical history was admitted to the hospital for severe erosive mucositis involving the lips, buccal mucosa, and hard and soft palate that limited her ability to eat and drink and required placement of a nasogastric tube for hydration and nutrition. She also had approximately one dozen targetoid and bullous pink papules and plaques on the bilateral palms, dorsal hands, chest, back, and limbs (Fig. 1), in addition to conjunctivitis and vaginal mucositis. She regularly took no medications other than desogestrel-ethinyl estradiol 0.15/0.03 mg daily. She had taken oral contraceptives for 7 years and had switched from norgestimate-ethinyl estradiol 0.25/0.035 mg to her current OCP 7 months prior.

A punch biopsy of a targetoid lesion on the left fourth digit revealed vacuolar alteration of the basal layer with dyskeratotic keratinocytes, beneath which laid a dense, band-like lymphocytic infiltrate consistent with lichenoid dermatitis, favoring mycoplasma-induced rash and mucositis or EM. Mycoplasma pneumonia IgG titers were positive, but mycoplasma pneumonia IgM titers were negative, suggesting a past infection that was likely unrelated. COVID-19, Epstein–Barr virus antibodies, hepatitis A, B, and C panels, and wound cultures were negative. HSV polymerase chain reaction of the oral lesions was negative as well. A complete blood count on admission was unremarkable. The patient was treated with methylprednisolone 80 mg intravenously for 3 days and azithromycin 250 mg daily for 5 days. By day 5, there was a gradual improvement of the cutaneous and orolabial lesions. The patient was subsequently discharged 2 days later with pain medication and dexamethasone oral swish and spit and required continued use of the nasogastric tube for a total of 10 days.

**Fig. 2.**
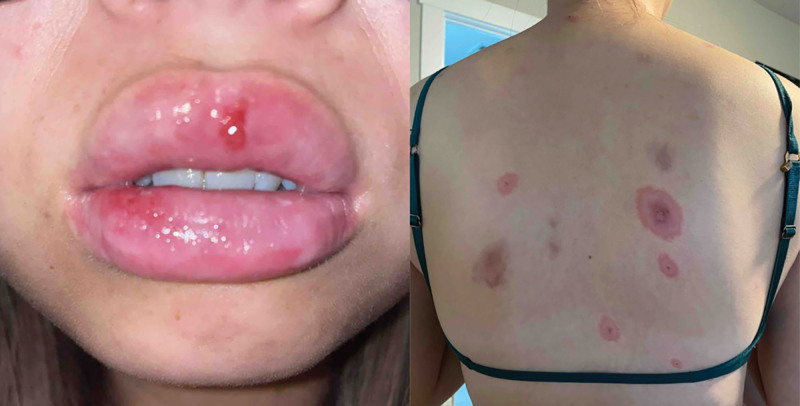
Mucosal erosions and targetoid papules flare despite being on empiric valacyclovir and 40 mg of prednisone.

Four months later, the patient presented to the clinic with recurrent oral ulcers and bullous targetoid lesions on her body similar to her previous hospital admission. HSV1/2 IgG and IgM titers were positive, and she was started on empiric valacyclovir 1 g daily for presumed HSV-associated bullous EM. A prednisone taper starting at 60 mg daily was initiated. She had a resolution within 1 week of starting the prednisone taper. However, she continued to flare once monthly despite empiric valacyclovir 1 g daily treatment (Fig. 2). Over the following approximately 18 months, the monthly oral and cutaneous eruptions failed prevention with dapsone 50 mg daily for greater than 3 months, azithromycin 250 mg 3 times weekly for 2 months, cyclosporine 125 mg twice daily (4.2 mg/kg) for 1 month, and methotrexate 15 mg by mouth once weekly for greater than 3 months. She then began adalimumab 80 mg subcutaneously once then 40 mg every 14 days. She completed 4 doses of adalimumab before the patient self-discontinued this treatment. At this same time, she self-discontinued her OCP. She had no further outbreaks of oral or cutaneous lesions after stopping the OCP and remained symptom-free 20 months later.

Bullous EM is an immune-driven mucocutaneous disorder characterized by rapid-onset, distinct target-like lesions.^1,2,4^ Numerous investigations have linked HSV to the initiation of recurrent EM.^1,3^ Successful use of continuous antiviral therapy has been demonstrated in the suppression of disease among some patients with recurrent EM.^5^ For patients who exhibit resistance to antiviral treatment, immunosuppressive agents are typically employed. While our patient with recurrent severe recalcitrant bullous EM may have had HSV-associated EM in the past given her positive HSV IgM antibodies, she continued to flare despite suppressive therapies and multiple courses of prednisone. The patient rapidly achieved sustained resolution with the cessation of OCPs.

A review of the literature revealed several reports of EM driven by endogenous progesterone, known as progesterone hypersensitivity or autoimmune progesterone dermatitis (summarized in Table 1).^6,7,11–15,17–19,21,22^ Progesterone hypersensitivity is characterized by a cyclical cutaneous eruption that may be clinically and histopathologically similar to EM, eczema, urticaria, or angioedema.^23,24^ Progesterone hypersensitivity is thought to be related to cyclical rises in endogenous progesterone, often appearing several days before menses in a fertile woman with a previous history of exogenous progesterone intake and sometimes in association with pregnancy.^23,25,26^

**Table 1:** Characteristics of patients with history of erythema multiforme due to endogenous and exogenous progesterone in the literature

Source	Age (yr), sex	Duration	Clinical features	Trigger	Localization	Failed treatment(s)	Effective treatment(s)	Effectiveness of treatment
Endogenous progesterone								
Çetinözman Aksoy et al.^6^	36, Female	2 yr	Bullous	Menses	Extremities, oral mucosa	Prednisone, spironolactone	Tamoxifen	Resolution
Cocuroccia et al.^7^	38, Female	3 yr	Bullous	Menses	Extremities, oral and genital mucosa	Acyclovir, prednisone, discontinuation of OCP	Tamoxifen	Resolution
George and Badawy^8^	38, Female	25 yr	Nonbullous	Menarche	Not specified	Antihistamines	OCP	Resolution
Irshad et al.^9^	29, Female	7 yr	Bullous	Menses	Extremities, back, oral mucosa	Diphenhydramine	Prednisone and topical clobetasol	Resolution
Kakarla and Zurawin^10^	15, Female	1 yr	Bullous	Menses	Eyelids, face, oral mucosa, fingers	None	Ethinyl estradiol and levonorgestrel	Resolution
Medeiros et al.^11^	42, Female	3 mo	Nonbullous	Pregnancy	Widespread	Prednisolone, antihistamines	Leuprorelin acetate, hysterectomy, and bilateral oophorectomy	Resolution
Nasabzadeh et al.^12^	22, Female	3 yr	Bullous	Menses	Upper eyelids, back, abdomen, extremities	Valacyclovir, drospirenone/ethinyl estradiol, topical corticosteroids	Danazol	Lost to follow-up
Pinto et al.^13^	23, Female	10 yr	Nonbullous	Menarche	Arms, legs, back, oral mucosa	Prednisone, colchicine, DDS	Thalidomide	Resolution
Rodenas et al.^14^	28, Female	5 yr	Nonbullous	Menses	Extremities, oral mucosa	Estrogens, tamoxifen, triptorelin	Oophorectomy	Resolution
Shelley et al.^15^	27, Female	5 yr	Bullous	Menses	Extremities, back	Sulfapyridine, systemic steroids, griseofulvin, antihistamines, sedation, topicals	Ethinyl estradiol, bilateral oophorectomy	Resolution
Toms Whittle et al.^16^	34, Female	8 yr	Bullous	Menses	Extremities, trunk	OCP	Buserelin	Resolution
Walling and Scupham^17^	38, Female	18 mo	Nonbullous	Discontinuation of OCP	Face	None	Cetirizine	Resolution
Warin^18^	26, Female	1 yr	Nonbullous	Menses	Extremities, oral and genital mucosa	Hydroxychloroquine, dapsone, cyclosporine	Nafarelin, azathioprine	Resolution
Wojnarowska et al.^19^	33, Female	10 wk	Nonbullous	Pregnancy	Hands, feet, trunk, oral mucosa	Ethinyl estradiol, prednisone, fresh frozen plasma infusions	Tamoxifen	Resolution
Exogenous progesterone								
Jawetz et al.^20^	18, Female	16 d	Nonbullous	Ethinyl estradiol and drospirenone	Oral mucosa	Acyclovir, diphenhydramine, amoxicillin clavulanate	Discontinuation of OCP, prednisone	Resolution
Suzuki et al.^21^	34, Female	3 wk	Nonbullous	Ethinyl estradiol and levonorgestrel	Extremities, trunk	Prednisolone, intravascular hydrocortisone sodium succinate	Discontinuation of OCP	Not reported

DDS, diaminodiphenyl sulfone; OCP, oral contraceptive pill.

In contrast, our patient had cyclical flares of bullous EM while taking exogenous desogestrel in her OCP, favoring drug-induced EM. There are only 2 other cases in the literature of patients who presented with EM due to an exogenous progesterone source and both of these presentations were nonbullous.^21^ Therefore, our case is the first reported incidence, to our knowledge, of recurrent bullous EM in association with OCPs with complete resolution upon discontinuation of OCP. This case highlights the importance of considering OCPs as a potential cause for drug-induced bullous EM.

## Conflicts of interest

None.

## Funding

None.

## Study approval

The authors confirm that any aspect of the work covered in this manuscript that has involved human patients has been conducted with the ethical approval of all relevant bodies.

## Author contributions

AG-S and TMG: Participated in research design, performance of the research, data analysis, and writing of the manuscript.

## Patient consent

Informed, written consent was received from all patients for whom photographs are present in the manuscript.
